# The association constant of 5′,8-cyclo-2′-deoxyguanosine with cytidine

**DOI:** 10.3389/fchem.2015.00022

**Published:** 2015-03-27

**Authors:** Amedeo Capobianco, Tonino Caruso, Sandra Fusco, Michael A. Terzidis, Annalisa Masi, Chryssostomos Chatgilialoglu, Andrea Peluso

**Affiliations:** ^1^Dipartimento di Chimica e Biologia, Università di SalernoFisciano, Salerno, Italy; ^2^Istituto per la Sintesi Organica e la Fotoreattività, Consiglio Nazionale delle RicercheBologna, Italy; ^3^Institute of Nanoscience and Nanotechnology, National Center for Scientific Research “Demokritos,”Athens, Greece

**Keywords:** DNA damage, cyclopurines, voltammetry, NMR, Watson-Crick complex

## Abstract

The association of 5′,8-cyclo-2′-deoxyguanosine (cdG), a DNA tandem lesion, with its complementary base cytosine has been studied by voltammetry and NMR in chloroform, using properly silylated derivatives of the two nucleobases for increasing their solubilities. Both voltammetric data and NMR titrations indicated that the Watson-Crick complex of cytidine with cdG is weaker than that with guanosine, the difference being approximately of one order of magnitude between the two association constants.

## Introduction

The level of distortion in DNA double helix is highly evaluated for the recognition and the repair of DNA lesions in cells. Among the various lesions that can be produced as consequence of metabolic processes and other external factors (oxidizing agents, drugs, ionizing, and non-ionizing irradiation, etc.) are the 5′,8-cyclo-2′-deoxypurines (cdP) where the base is covalently connected with the sugar with an extra carbon-carbon bond apart from the usual glycosidic bond (Chatgilialoglu et al., 2011). Both cdA and cdG lesions exist as 5′R- and 5′S-diastereomers. The structural changes that a C5′–C8 bond causes compared to natural purine nucleosides and the consequential distortion at the local DNA sequence, have been found efficient enough for the selective activation of the complex nucleotide excision repair (NER) apparatus in cells for restoring the damage. By the direct comparison of all four cdP lesions in NER efficiency studies, it has been reported that cdA and cdG are excised with similar yields while the configuration of the C5′ influences the repair yield with the 5′R lesions being 2 times more efficiently repaired (Kropachev et al., 2014).

The cdP are lesions observed among the DNA modifications and identified in mammalian cellular DNA *in vivo* (Chatgilialoglu et al., 2011). Recent studies reported cyclopurine lesions as reliable oxidative stress biomarkers in animal models (Wang et al., 2011; Mitra et al., 2012).

NMR studies integrated by molecular dynamics simulations have shown that replacing a deoxyguanosine (dG) with a 5′S-cdG in a B-DNA dodecamer results in perturbations of the helical twist and base pair stacking at the lesion sites (Huang et al., 2011). A similar behavior was observed in duplexes containing 5′S-cdA in place of dA (Zaliznyak et al., 2012). In both cases thermodynamic destabilization of the damaged duplexes was observed, as testified by the lowering of the melting point of the modified sequence with respect to unmodified DNA, amounting e.g., to 9°C for a double stranded dodecamer containing a single 5′S-cdG (Huang et al., 2011).

Molecular dynamics simulations highlighted that both the 5′R-cdA and 5′R-cdG lesions are significantly more distorting than the respective 5′S diastereomers, thus the different efficiency of NER was traced back to the larger stacking impairment induced by 5′R isomers (Kropachev et al., 2014). However, the same lowering of melting temperature was observed for modified 17-mers double stranded oligonucleotides containing cdG and differing only for the absolute configuration at the 5′ carbon.

It is worth mentioning that the bulkiness of a lipophilic derivative of 5′S-cdG did not inhibit the self-assembly of the lesions in G-quartets as revealed by NMR experiments. However, the type of the supra-molecular organization was identified as all-*anti-*G-quadruplex, realizing the first exception to the all *syn* conformation as a critical factor for a G_4_ self-assembly in non-aqueous medium (Pieraccini et al., 2014).

Due to their multidisciplinary importance, further exploration of the purine 5′,8-cyclonucleoside behavior in respect with its complementary base was investigated herein. Particularly, the lack of correlation of the melting temperatures with the extent of chain distortion and NER efficiencies indicated the existence of additional effects, such as the interaction of cdG with its complementary nucleoside, that may play a role in the thermodynamic destabilization of helices containing cyclopurines (Pande et al., 2012). Differential pulse voltammetry revealed to be a very powerful technique for investigating inter-base interactions between complementary nucleobases (Caruso et al., 2005, 2007), (Capobianco et al., 2013b; Capobianco and Peluso, 2014) thus, aiming at investigating the peculiar interaction between cdG and cytidine, we have carried out a voltammetric and NMR study of lipophilic derivatives of the 5′S-cdG or 5′R-cdG with 2′-deoxycytidine in chloroform, a solvent in which the nucleosides mainly form H-bond complexes, mimicking the hydrophobic core of DNA (Kyogoku et al., 1967; Williams et al., 1987).

## Materials and methods

To increase solubility in CHCl_3_ and prevent interference of hydrogen bonding by ribose hydroxyls, 2′-deoxycytidine was derivatized with tert-butyldimethylsilyl groups on the 2-deoxyribose unit to yield 3′,5′-bis-*O*-(*tert*-butyldimethylsilyl)-2′-deoxycytidine (dC′, Scheme I) (Caruso et al., 2005). (5′S)- and (5′R)-3′,5′-bis-*O*-(*tert*-butyldimethylsilyl)-5′,8-cyclo-2′-deoxyguanosine (*S*cdG′, *R*cdG′) were prepared by following the reported procedure (Terzidis and Chatgilialoglu, 2013).

**Scheme I:**
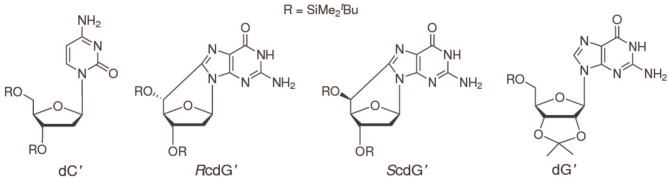


^1^H-NMR spectra were recorded in CDCl_3_ by Bruker Advance 600 MHz. Differential pulse and cyclic voltammetry measurements were performed at room temperature in chloroform (Spectroscopic grade, Sigma-Aldrich), scan rate = 100 mV/s, by Autolab PGSTAT 302N potentiostat-galvanostat. A three-electrode cell configuration was adopted, Pt bars have been employed for quasi-reference and counter electrodes and a glassy carbon electrode (Metrohm) was used as working electrode; a positive feedback was applied to compensate for ohmic drop. The potential of the quasi-reference electrode was calibrated against the ferrocenium/ferrocene couple (Fc^+^/Fc) (Capobianco et al., 2009; Bard and Faulkner, 2001). Tetrabutylammonium perchlorate (electrochemical grade, Sigma-Aldrich) 0.02 M was used as the supporting electrolyte. Solutions were deaerated by bubbling nitrogen before each experiment.

## Results and discussion

**Figure 1:**
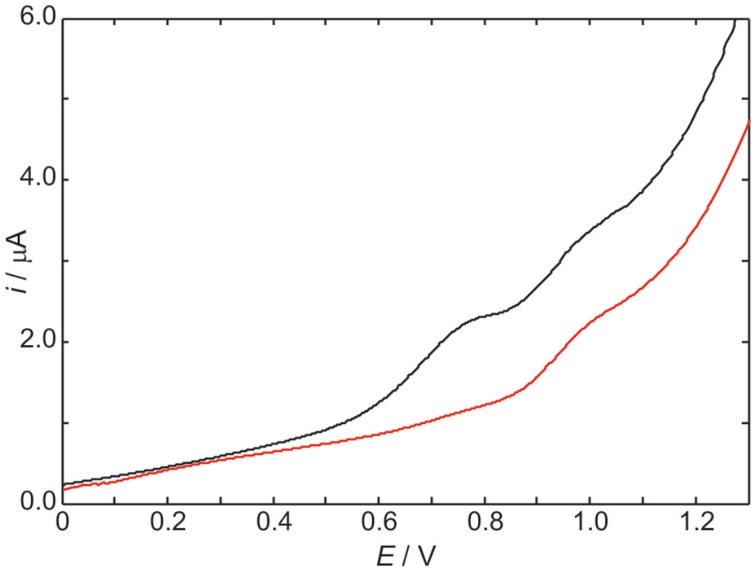
**Differential pulse voltammograms in CHCl_3_ 20 mM TBAP of 1.0 mM *S*cdG′ (red curve) and 1.0 mM *S*cdG′: 1.0 mM dC′ (black curve)**. Scan rate, 100 mV/s.

The differential pulse voltammogram of a 1.0 mM solution of *S*cdG′ (see Scheme I) in chloroform is reported in Figure 1 (red curve); an irreversible oxidation peak is observed at 0.95 V vs. Fc^+^/Fc, a potential very close to the one (0.91 V) observed for the oxidation of dG′ (Caruso et al., 2005) thus indicating that the formation of the C8–C5′ covalent bond does not constitute a severe perturbation of the π system of guanine, as expected.

The voltammogram of an equimolar solution of *S*cdG′ and dC′ (Figure 1, black curve) exhibits two voltammetric signals, one occurring at the same potential observed for solutions containing only 5′,8-cyclo-2′-deoxyguanosine, which can be safely assigned to the fraction of free *S*cdG′ in solution, the other peaked at 0.77 V. Since dC′ oxidation does not show any signal in the allowed potential window in chloroform, the lower potential peak is assigned to the 1:1 *S*cdG′:dC′ H-bond complex, in analogy with the case of guanosine and cytidine nucleosides (Caruso et al., 2005). Indistinguishable results were obtained for the *R* diastereoisomer and its 1:1 complex with dC′.

The stabilization of *S*cdG′^+ ·^ (or *R*cdG′^+ ·^) radical cation by cytidine in chloroform amounts to 0.18 eV, a value considerably lower than that observed for the pairing of dG′ with dC′ (0.34 eV) and even smaller than the oxidation potential shift of adenosine due to the formation of adenosine:thymidine complexes in chloroform (0.28 eV) (Caruso et al., 2005, 2007).

The above result has relevant consequences on binding energies. Consider *A* and *B* species forming the *AB* complex; the association and oxidation Gibbs free energies are defined as:

(1) $$\Delta G_{A\ldots B}^{ass}=G_{AB}-G_{A}-G_{B}$$

and

(2) $$\Delta G_{A}^{ox}=G_{A+}-G_{A}$$

By using Equations (1, 2), and assuming that *A* monomer holds the whole positive charge in the singly ionized complex {AB}^+^ we have:

(3) $$\begin{array}{l} \Delta G_{A}^{ox}-\Delta G_{AB}^{ox}=\Delta G_{A\ldots B}^{ass}\;-\;\Delta G_{A+\ldots B}^{ass}\;=\;\Delta H_{A\ldots B}^{ass}\\ \;-\Delta H_{A+\ldots B}^{ass}\;-\;T\left(\Delta S_{A\ldots B}^{ass}\;-\;\Delta S_{A+\ldots B}^{ass}\right) \end{array}$$

Since

(4) $$△H_{A\ldots B}^{ass}\;\approx \;△E_{A\ldots B}^{ass}\;=\;E_{A\ldots B}^{bind}$$

where *E*^*bind*^_A… B_ is the binding energy in the *AB* complex, if we make the reasonable approximation:

(5) $$△S_{A\ldots B}^{ass}\;\approx \;△S_{A+\ldots B}^{ass}$$

then Equation (3) becomes:

(6) $$△G_{A}^{ox}\;-\;△G_{AB}^{ox}\;\approx \;E_{A\ldots B}^{bind}\;-\;E_{A+\ldots B}^{bind}$$

Application of Equation (6) in which *B* is dC′ and *A* is dG′, *S*cdG′ or *R*cdG′ shows that cdG′:dC′ and dG′:dC′ complexes are characterized by different interaction energies both in the neutral and in the charged state. To evaluate the binding energy of the *S*cdG′:dC′ complex, we have thus resorted to NMR titration experiments.

**Figure 2:**
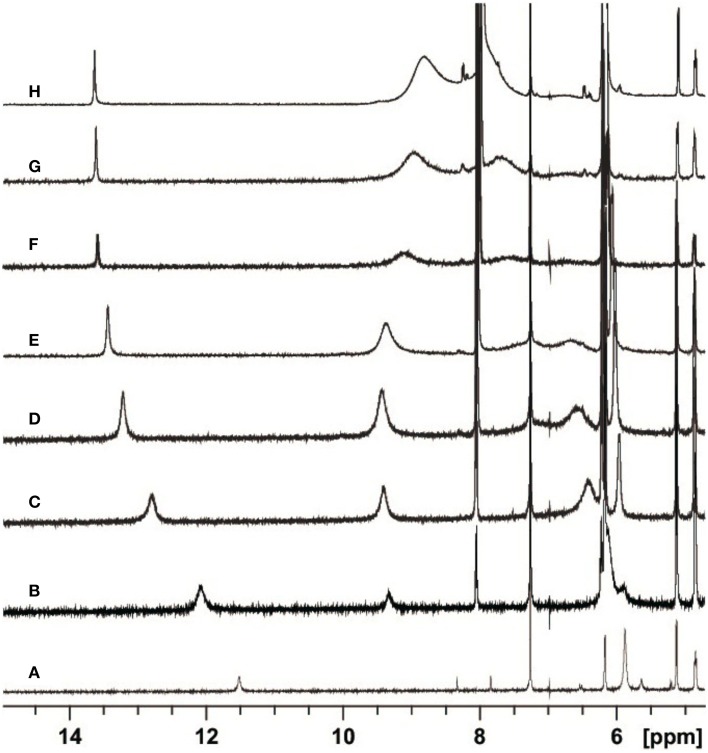
**Partial H-NMR (600 MHz) spectra of 3.6 mM *S*cdG′ at *T* = 22 C in the presence of 0 (A), 1.7 (B), 4.7 (C), 8.7 (D), 13.0 (E), 25.0 (F), 36.7 (G), and 68.3 (H) mM dC′ in CDCl_3_**.

In Figure 2 the most interesting region of the NMR spectra of solutions containing dC′ and *S*cdG′ in different ratios is reported. As the concentration of dC′ increases, the imino N1–H proton shift of *S*cdG′ increases from 11.5 ppm to 13.7 ppm, indicating that the Watson-Crick complex, which is by far the most stable motif for G:C association (Abo-Riziq et al., 2005) is actually established in CHCl_3_, as also observed in modified DNA (Huang et al., 2011).

**Figure 3:**
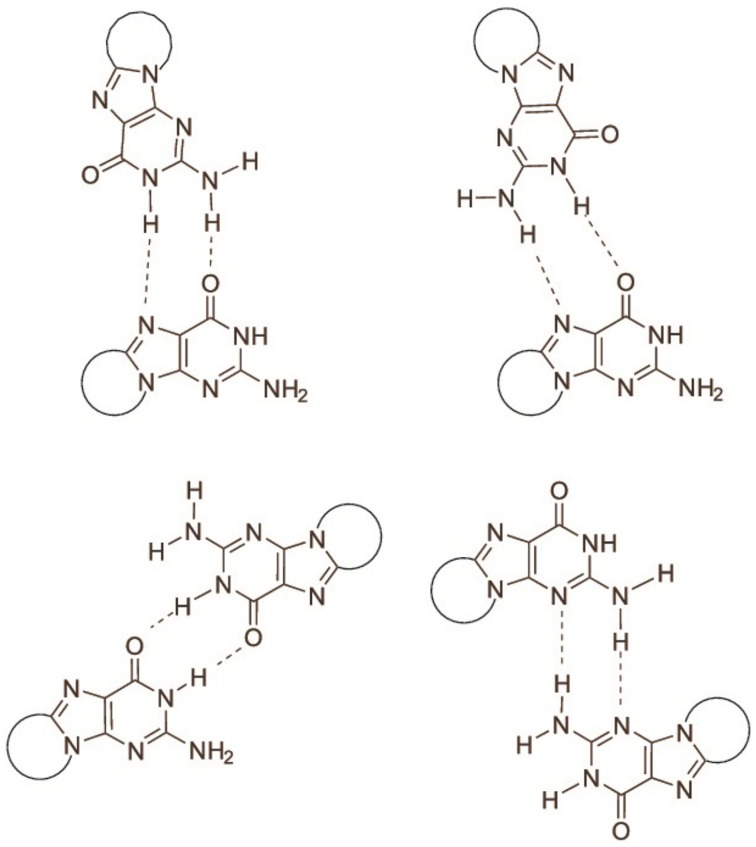
**Association modes of 5′,8-cyclo-2′-deoxyguanosine dimers**.

The determination of the association equilibrium constant for the *S*cdG′:dC′ Watson-Crick complex is not an easy task since many competitive processes may occur in solution. It is indeed well-known that guanosine self-associates in non-protic solvents, leading to at least four different dimeric complexes, see Figure 3, each of which is characterized by a different self-association constant. Moreover, hetero-association between guanine and cytosine is accompanied by additional, more intricate hydrogen-bonded complexes, such as trimers (cytosine:guanine_2_) and tetramers [(cytosine:guanine)]_2_ in weakly polar solvents (Williams et al., 1989; Sartorius and Schneider, 1996). Because of that, use of non-linear least-squares fits, explicitly considering the formation of self-association dimers GG and CC, in addition to the G-C base pair, are needed to obtain reliable association constants (Sartorius and Schneider, 1996) whereas more conventional treatment have only allowed to obtain rough estimates of both the self-association constant of guanine and the hetero-association constant with cytosine (Kyogoku et al., 1969).

It was therefore necessary to determine the self-association constant of cycloguanosine in CDCl_3_ first in order to obtain a reliable estimate of the hetero-association constant of cycloguanosine with cytidine.

By following the chemical shift of the imino proton at increasing concentrations of cycloguanosine and using the standard equation (hereafter we further simplify notation, by indicating dC′ as C and *S*cdG′ as G in the following equations):

(7) $$\delta_{obs}=\delta_{G}\;+\;\frac{\delta_{G2}-\delta_{G}}{4k_{D}{\left[G\right]}_{0}}\left(4k_{D}{\left[G\right]}_{0}+1-\;\sqrt{8k_{D}{\left[G\right]}_{0}+1}\right)$$

where *k_D_* is the dimerization constant, *δ_G_, δ_G2_* the shifts of the imino protons in G and G_2_, respectively and [*G*]_0_ is the analytical concentration of cycloguanosine (Davies et al., 1996), we have obtained *k_D_* ≈ 10^3^ at 25°C.

The 1:1 model for the G:C association should be reliable only in the presence of an excess of cytidine, because in that case both the self-association of cycloguanosine and the formation of trimers and tetramers are disfavored. Moreover self-association of cytidine does not constitute a problem, because it is known to be negligible in chloroform (Kyogoku et al., 1969; Sartorius and Schneider, 1996).

The observed chemical shift of the imino proton when the simultaneous equilibria for the self and the hetero-association are considered, is given by:

(8) $$\delta_{obs}=\left(1-\alpha -2\beta \right)\delta_{G}+\alpha \delta_{GC}+2\beta \delta_{G2}$$

where α and β are the hetero and self- association degrees, respectively and are related by:

(9) $$\beta =\frac{\alpha^{2}{\left[G\right]}_{0}k_{D}}{k_{A}^{2}{\left({\left[C\right]}_{0}-\alpha {\left[G\right]}_{0}\right)}^{2}}$$

*k_A_* being the hetero-association constant. In the case of a large excess of cytidine, *α* may be approximated as:

(10) $$\alpha =\frac{{\left[C\right]}_{0}+{\left[G\right]}_{0}+k_{A}^{-1}-\sqrt{{\left({\left[C\right]}_{0}+{\left[G\right]}_{0}+k_{A}^{-1}\right)}^{2}-4{\left[G\right]}_{0}\left(1-\frac{2k_{D}}{k_{A}^{2}{\left[C\right]}_{0}}\right){\left[C\right]}_{0}}}{2{\left[G\right]}_{0}\left(1-\frac{2k_{D}}{k_{A}^{2}{\left[C\right]}_{0}}\right){\left[C\right]}_{0}}$$

**Figure 4:**
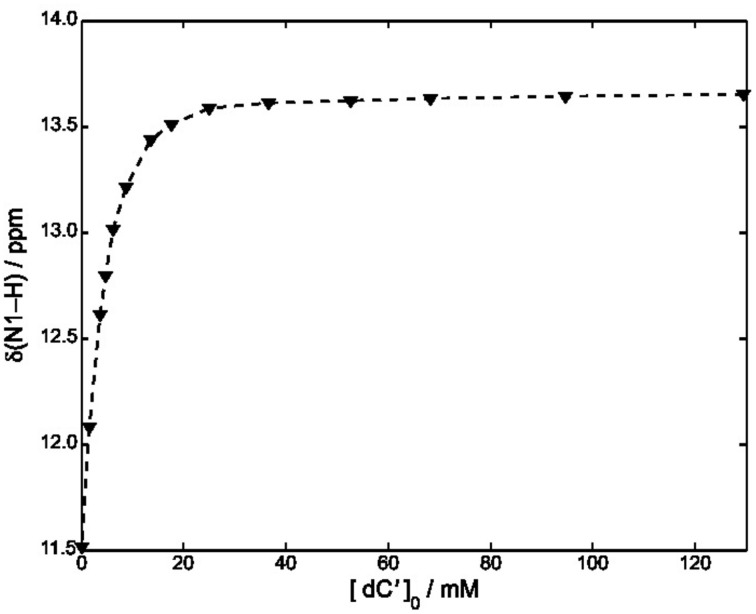
**Plot of δ (N1–H) vs. [dC′] in CDCl_3_ at 22°C**. The concentration of *S*cdG′ was maintained constant at 3.6 mM. The dashed curve has been obtained by using Equation (11) and fitting all the experimental points.

Equations (8–10) have been used to fit the NMR data of Figure 4, yielding *k_A_* = 1.4· 10^3^ M^−1^ at 22°C.

A slightly lower estimate, *k_A_* = 7· 10^2^ *M^−1^* was obtained by fitting all the points of Figure 4 and neglecting the self-association constant of cycloguanosine according to the well-known equation:

(11) $$\delta_{obs}=\delta_{G}+\frac{\delta_{GC}-\delta_{G}}{2{\left[G\right]}_{0}}\left({\left[C\right]}_{0}+{\left[G\right]}_{0}+k_{A}^{-1}-\sqrt{{\left({\left[C\right]}_{0}+{\left[G\right]}_{0}+k_{A}^{-1}\right)}^{2}-4{\left[G\right]}_{0}{\left[C\right]}_{0}}\right)$$

We have also considered a further fitting model, by using only the points for which [*dC′*]_0_ > 10 [*ScdG′*]_0_ (Fielding, 2000) in conjunction with the NMR version of the Benesi-Hildenbrand equation (Mathur et al., 1963). A similar value was obtained for the hetero-association constant, namely *k_A_* = 1· 10^3^M^−1^.

Independent of the adopted approximation, *k_A_* is estimated to be one order of magnitude less than that obtained for the hetero-association of guanosine and cytidine in chloroform (2.1·10^4^ M^−1^), Caruso et al. (2007) thus showing that the interaction of dC′ with *S*cdG′ is weaker than that with dG′. Moreover the equilibrium constant for the formation of the Watson-Crick complex appears to be slightly lower than that for the homo-association of guanosine in chloroform thus confirming that all the equilibria must be considered.

## Conclusions

The interactions of *S*cdG′ and *R*cdG′, two important DNA tandem lesions (Scheme I), with their complementary base have been studied by NMR spectroscopy and differential pulse voltammetry in chloroform solution, a solvent where guanosine and cytidine are known to form the Watson-Crick hydrogen bonded complex. The oxidation potentials of *S*cdG′ and *R*cdG′ are almost the same as the undamaged nucleoside, whereas the oxidation potentials of their Watson-Crick complexes with cytidine are significantly higher (0.16 eV) than that of dG′:dC′. Those somewhat intriguing results suggest that the interaction energies of the damaged nucleosides with their complementary one differ from that of guanosine. In line with that expectation, the equilibrium association constant for the *S*cdG′:dC′ complex, determined by NMR titrations in different ways, both by explicitly considering the existence of the simultaneous cdG′ self-association equilibrium and by working in conditions of large cytosine excess, is found to be ca. 10^3^ M^−1^, about one order of magnitude less than its counterpart for undamaged nucleobase. No significant changes with respect to dG′:dG′ have been found for the self-association *S*cdG′:*S*cdG′ equilibrium constant, obtained under the simplified assumptions that either all self-association modes possess the same equilibrium constant or that one association mode is predominant over all the others. In summary the results reported here, although far from being exhaustive, suggest that other factors besides helical distortions are effective for thermodynamic destabilization of DNA tracts containing cycloguanosine.

### Conflict of interest statement

The authors declare that the research was conducted in the absence of any commercial or financial relationships that could be construed as a potential conflict of interest.
